# Supplementary material for: Low-Molecular-Weight Fucoidan as Complementary Therapy of Fluoropyrimidine-Based Chemotherapy in Colorectal Cancer
Source: Int J Mol Sci. 2021 Jul 27;22(15):8041. doi: 10.3390/ijms22158041 (PMC8347453; doi:10.3390/ijms22158041)
Supplement: Supplementary file 1 [file ijms-22-08041-s001.zip › ijms-1282817-Supplementary.pdf]

S1A

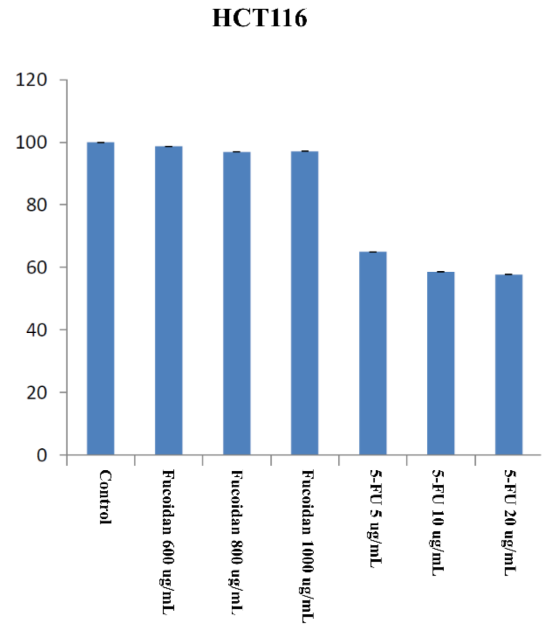

S1B

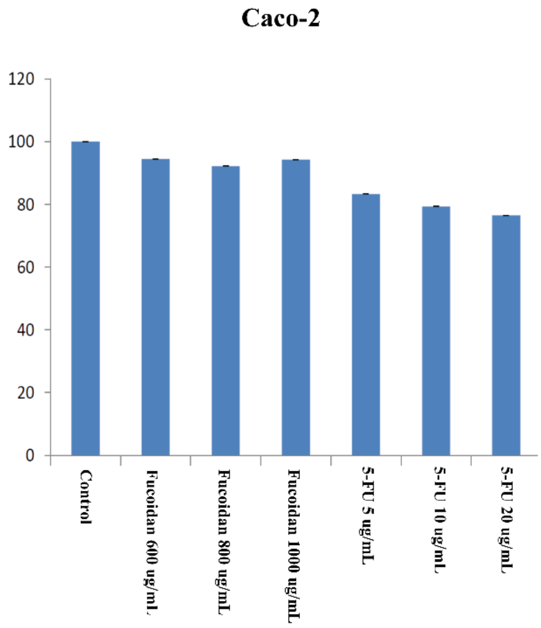

Figure S1. Effects of LMWF and 5-fluorouracil (5-FU) on viability after treatment with LMWF (three different doses: 600, 800, and 1000 µg/mL) and 5-FU (three different doses: 5, 10, and 20 µg/mL). (**A**) HCT116 cells. (**B**) Caco2 cells.
